# Pyomelanin-Producing *Brevundimonas vitisensis* sp. nov., Isolated From Grape (*Vitis vinifera* L.)

**DOI:** 10.3389/fmicb.2021.733612

**Published:** 2021-10-14

**Authors:** Lingmin Jiang, Doeun Jeon, Jueun Kim, Chul Won Lee, Yuxin Peng, Jiyoon Seo, Ju Huck Lee, Jin Hyub Paik, Cha Young Kim, Jiyoung Lee

**Affiliations:** ^1^Korean Collection for Type Cultures, Biological Resource Center, Korea Research Institute of Bioscience and Biotechnology (KRIBB), Jeongeup, South Korea; ^2^Department of Chemistry, Chonnam National University, Gwangju, South Korea; ^3^International Biological Material Research Center, Korea Research Institute of Bioscience and Biotechnology (KRIBB), Daejeon, South Korea

**Keywords:** phylogenetic analysis, strain GR-TSA-9^T^, endophytic bacterium, natural pigments, melanin production

## Abstract

A novel endophytic bacterial strain, designated GR-TSA-9^T^, was isolated from surface-sterilized grape (*Vitis vinifera* L.). 16S rRNA gene sequence analyses showed that the isolate was grouped within the genus *Brevundimonas*, displaying the highest similarity with *Brevundimonas lenta* DS-18^T^ (97.9%) and *Brevundimonas kwangchunensis* KSL-102^T^ (97.8%) and less than 97.5% similarity with other members of *Brevundimonas*. The strain GR-TSA-9^T^ was a gram negative, rod shaped, facultatively anaerobic, catalase and oxidase positive, and motile bacterium. Its growth occurred at 10–37°C (optimally 25–30°C), at pH 7.0–8.0, and in NaCl 0–1% (optimally 0%). It contained ubiquinone-10 as a respiratory quinone, and the major cellular fatty acids (>10% of the total) were C_16:0_ (14.2%) and summed feature 8 (C_18:1_ω7*c* and/or C_18:1_ω6c, 65.6%). The polar lipids present in the strain were phosphoglycolipids, phosphatidylglycerol, 1,2-di-*O-*acyl-3-O-[d-glucopyranosyl-(1→4)-α-d-glucopyranuronosyl]glycerol, and unidentified lipids (L1, L2, and L4). The strain had one 2,976,716bp circular chromosome with a G+C content of 66.4%. The digital DNA–DNA hybridization value between strain GR-TSA-9^T^ and *B. lenta* DS-18^T^ was 20.9%, while the average nucleotide identity value was 76.7%. In addition, the dDDH and ANI values to other members in this genus, whose genome sequences are available, are less than 21.1 and 77.6%. Genome annotation predicted the presence of some gene clusters related to tyrosine degradation and pyomelanin formation. Strain GR-TSA-9^T^ produced a brown melanin-like pigment in the presence of L-tyrosine-containing media. The highest pigment production (0.19g/L) was observed in tryptic soy broth with 1.0mg/ml L-tyrosine at 25°C for 6days of culture. Biophysical characterization by ultraviolet (UV)–visible spectroscopy, Fourier-transform infrared spectroscopy, and electrospray ionization mass spectrometry confirmed that the pigment was pyomelanin. Additionally, melanized GR-TSA-9^T^ cells could protect the cells against UVC exposure. The phylogenetic, genomic, phenotypic, and chemotaxonomic features indicated that strain GR-TSA-9^T^ represents a novel melanin-producing species of *Brevundimonas*. The type strain was GR-TSA-9^T^ (KCTC 82386^T^=CGMCC 1.18820^T^).

## Introduction

The bacterial genus *Brevundimonas* was reclassified from the genus *Pseudomonas* as a member of the family *Caulobacteraceae* based on a polyphasic approach; type strains *Brevundimonas diminuta* ATCC 11568^T^ and *Brevundimonas vesicularis* ATCC 11426^T^ were proposed at the same time (Segers et al., 1994). Several *Caulobacter* species, such as *C. bacteroides*, *C. intermedius*, *C. subvibrioides*, *C. variabilis*, and *Caulobacter* subspecies, such as *C. henricii* subsp. *aurantiacus*, *C. subvibrioides* subsp. *albus*, as well as *Mycoplana* species, such as *Mycoplana bullata* (Abraham et al., 1999), were subsequently transferred to the genus *Brevundimonas*. As of April 2021, 32 species have been reported in this genus.^1^ Members of the genus *Brevundimonas* survive in diverse environments, including deep-subsea floor sediment (Tsubouchi et al., 2013), different soil sources (Yoon et al., 2006, 2007; Pham et al., 2016), the sea (Fritz et al., 2005), activated sludge (Ryu et al., 2007; Qu et al., 2019; Lee et al., 2020), humans (Sofer et al., 2007; Estrela and Abraham, 2010), aquatic habitats (Abraham et al., 2010), and floor sediments (Tsubouchi et al., 2013, 2014). The genus *Brevundimonas* has been reported to be a growth promoter in agriculture (Singh et al., 2016; Naqqash et al., 2020) and a bioremediation tool for the removal of copper (Rathi and Yogalakshmi, 2021), dimethachlon (Zhang et al., 2020), and arsenic toxicity (Singh et al., 2016). Furthermore, some species of this genus were found to be opportunistic pathogens isolated from clinical samples (Chandra et al., 2017; Ryan and Pembroke, 2018). Members of this genus are characterized as gram-negative, facultatively anaerobic or aerobic, rod-shaped, oxidase- and catalase-positive, non-fermenting bacteria with a relatively high DNA G+C content (Wang et al., 2012; Qu et al., 2019; Lee et al., 2020). Ubiquinone 10 (Q-10) is a major isoprenoid quinone. Interestingly, *Brevundimonas* sp. SGJ was reported to produce L-dihydroxylphenylalanine (DOPA) melanin, which is predominantly an indolic polymer that is widely used in cosmetics, agriculture, and medicine (Surwase et al., 2012a,b, 2013).

Melanin, a high-molecular-weight polymer, is a ubiquitous natural pigment widely encountered in all organisms. Most microbial melanins are formed through the transformation of either tyrosine (3, 4-DOPA pathway) or malonyl-coenzyme A (dihydroxynaphthalene pathway), which is facilitated by different sets of enzymes. The melanin precursor tyrosine is converted to L-DOPA by tyrosinase (EC 1.14.18.1) and laccase (EC 1.10.3.2; Pavan et al., 2020). The second pathway for melanin synthesis is, endogenously produced malonyl-CoA is catalyzed by polyketide synthases and converted to 1,3,6,8-tetrahydroxynaphthalene and polymerized to dihydroxynaphthalene melanin (Pavan et al., 2020). The third pathway for melanin synthesis is the homogentisate pathway, wherein 4-hydroxyphenylpyruvate dioxygenase (*hppD*; EC 1.13.11.27) is the most critical enzyme for transfer to homogentisic acid (HGA) melanins (or pyomelanin), and the *hppD* gene plays a vital role in the HGA pathway (Singh et al., 2018). In the present study, a new strain, GR-TSA-9^T^, isolated from grapes was characterized as a novel species of the genus *Brevundimonas* based on the results obtained from a polyphasic taxonomic study. This strain is of interest because it can synthesize melanin. Whole-genome analysis was used to investigate the underlying pathways involved in pyomelanin biosynthesis mediated by the homogentisate pathway. Here, we present a novel species of the genus *Brevundimonas*, *B. vitisensis* sp. nov., with respect to taxonomy, genome analysis, and identification of pyomelanin production.

## Materials and Methods

### Bacterial Strains

Grapes were collected from Jeongeup (35°424.176' and 126°547.308'), and 5g grape was surface-sterilized with 1.05% sodium hypochlorite for 10min, followed by rinsing five times with sterile distilled water. After grinding in 20ml sterile water, 100μl of each sample was spread onto tryptic soy agar (TSA) medium and incubated at 25°C for 4days. Single colonies were obtained and subsequently streaked on fresh TSA medium. One grayish white, circular, smooth, opaque, and flat isolate, designated as GR-TSA-9^T^, was selected for further taxonomic study and preserved in sterile skimmed milk (10%, w/v) at −80°C. The strain was deposited at the Korean Collection for Type Cultures (KCTC) and the China General Microbiological Culture Collection (CGMCC) as KCTC 82386^T^ and CGMCC 1.18820^T^, respectively. Unless otherwise stated, the cells were grown on TSA medium at 25°C for 5days for subsequent tests.

### Phenotypic and Biochemical Characterization

Cell morphology was observed using scanning electron microscopy (Quanta 250 FEG) at the KRIBB Microscopy Core Facility. A Gram staining kit (Difco) was used according to the manufacturer’s instructions. Cells were grown on soft King B (peptone 20g/L, MgSO_4_7H_2_O 1.5g/L, and K_2_HPO_4_ 1.5g/L) medium containing agar (0.3, 0.5, and 1.5% w/v) for 5days to test swimming, swarming, and twitching motilities (Belgini et al., 2014). To determine catalase activity, bubble production was observed after adding 3% (v/v) hydrogen peroxide solution to fresh cells (Lee and Jeon, 2017). An oxidase reagent kit (bioMérieux) was used to determine oxidase activity. Growth of strain GR-TSA-9^T^ was observed using different media, namely nutrient agar (NA, beef extract 3g/L, peptone 5g/L, and agar 15g/L), potato dextrose agar (PDA), Luria–Bertani agar (LB), TSA, marine agar 2216 (MA), reasoner’s 2A agar (R2A), and reinforced clostridial agar (RC). Various temperatures of 4, 10, 15, 20, 25, 30, 37, 40, 45, and 60°C were assessed for optimal growth. Tryptic soy broth (TSB) with different pH values ranging from 3.0 to 12.0 was prepared using 10mM Tris–HCl (Hwang et al., 2018) and adjusted with 1N HCl and NaOH, while salt concentrations ranging from 0 to 15% (w/v; 1% concentration increments; Lee et al., 2019) were prepared to determine the optimal pH and salt concentration, respectively. Optical density at 600nm (OD_600_) was measured using an Optizen POP UV/VIS spectrophotometer (Optizen) to monitor the pH range and NaCl tolerance. The anaerobic growth was tested by incubating strain GR-TSA-9^T^ on TSA medium in an anaerobic chamber (Coy Scientific) filled with 86% N_2_, 7% CO_2_, and 7% H_2_ at 25°C for 7days. Other biochemical features, such as enzymes, were tested using API 20NE (bioMérieux) or API ZYM with NaCl 0.85% medium (bioMérieux) according to the manufacturer’s protocol.

### 16S rRNA Gene Sequence Analysis

The almost-complete 16S rRNA gene sequence of strain GR-TSA-9^T^ was amplified by polymerase chain reaction using the universal primers 27F and 1492R (Lane, 1991), and sequencing was performed using primers 27F/1492R and 518F (5'-CCAGCAGCCGCGGTAATACG-3') and 800R (5'-TACCAGGGTATCTAATCC-3'). The nearly full-length 16S rRNA gene was 1394 nucleotides in length and was compared to the corresponding sequences of the type strains obtained from the EzBioCloud server (Yoon et al., 2017).^2^ BioEdit (V.7.0.5) was used to perform multiple sequence alignments using all validly published members of the family *Caulobacteraceae*. Phylogenetic trees were constructed using the neighbor-joining (NJ), minimum evolution (ME), and maximum likelihood (ML) algorithms with 1,000 bootstrap iterations in the Molecular Evolutionary Genetics Analysis version 7.0 program (Kumar et al., 2016). Evolutionary distances for the NJ, ME, and ML analyses were calculated using Kimura’s two-parameter model.

### Genomic Sequencing and Annotation

Genomic DNA of strain GR-TSA-9^T^ was isolated using a genomic DNA purification kit (MGmed, Republic of Korea). The quantity and quality of the extracted genomic DNA were measured using PicoGreen and Nanodrop (ratio A260/A280). Whole-genome sequencing was performed at the Macrogen facility (Macrogen, Korea) on the PacBio RSII (Pacific Biosciences, Inc.) and the Illumina sequencing platform and assembled using SMRT Portal (version 2.3) *de novo* assembler. Potential contamination of the genome sequence was verified using ContEst16S (Lee et al., 2017).^3^ The assembled sequences were annotated using the National Center for Biotechnology Information Prokaryotic Genome Annotation Pipeline (PGAP) and the Rapid Annotation Subsystem Technology (RAST) server with the SEED database (Tatusova et al., 2016). Metabolic pathways were reconstructed using BlastKOALA based on the Kyoto Encyclopedia of Genes and Genomes pathway database (Kanehisa et al., 2016). The digital DNA–DNA hybridization (dDDH) and average nucleotide identity (ANI) values between strain GR-TSA-9^T^ and several closely related strains were calculated using the Genome-to-Genome Distance Calculation web server^4^ using the BLAST method and recommended formula 2 (Meier-Kolthoff et al., 2013) as well as an ANI calculator.^5^ The orthoANI values among the closely related strains were calculated using the standalone Orthologous Average Nucleotide Identity (OAT) software (Lee et al., 2016). Whole-genome sequences of closely related strains publicly available at NCBI GenBank were collected, and a whole-genome-based phylogenetic tree was constructed using the up-to-date bacterial core gene set and pipeline as described by Na et al. (2018). Briefly, the 92 core genes were extracted from genomes using Prodigal v2.6.3 (Hyatt et al., 2010) and hmmsearch v3.1b2 (Eddy, 2011). Predicted coding sequences (CDS) of 92 core genes were aligned by using Multiple Alignment using Fast Fourier Transform (MAFFT) for alignments (ver. 7.310; Katoh and Standley, 2013). Then, the phylogenomic tree was inferred by using the FastTree (Price et al., 2010) and viewed using MEGA v7.0 (Kumar et al., 2016). The branch support inference was based on 100 nonparametric bootstrap replicates, and the branch supports for the phylogenomic tree were evaluated using gene support index (GSI).

### Chemotaxonomic Characterization

For fatty acid methyl ester analysis, cells grown for 3days on TSA were extracted according to the instructions of the standard MIDI (Sherlock Microbial Identification System version 6.0), and cellular fatty acid content was analyzed by gas chromatography (Model 6890 N; Agilent) using the Microbial Identification software package (Sasser, 2006). Freeze-dried cells (100mg) were used to extract isoprenoid quinones using a chloroform-to-methanol (2:1, v/v) mixture, followed by analysis using thin-layer chromatography as described by Collins et al. (1980). Subsequent analysis was performed using reverse-phase high-performance liquid chromatography with ultraviolet (UV) absorbance detection at 270nm. Polar lipids were extracted from 100mg of freeze-dried cells with a chloroform/methanol mixture (1:2, v/v) and then identified by two-dimensional thin-layer chromatography on Kieselgel 60F254 plates (silica gel, 10cm×10cm; Merck). In addition, 0.2% ninhydrin (Sigma-Aldrich), α-naphthol, molybdenum blue (Sigma-Aldrich), 4% phosphomolybdic acid reagent, and Dragendorff’s solution were sprayed onto the plates to detect amino group-containing lipids, sugar-containing lipids, phosphorus-containing lipids, total lipids, and quaternary nitrogen-containing lipids, respectively.

### Production and Purification of the Microbial Pigment

The melanin-producing ability of strain GR-TSA-9^T^ was confirmed by growth on TSA medium supplemented with 0 and 10mg/ml of l-tyrosine followed by incubation at 25°C. Production was performed in TSB consisting of 0, 0.2, 0.4, 0.6, 0.8, and 1.0mg/ml l-tyrosine, and after inoculation with 1ml of the cell suspension (OD_600_=1.0) in 100ml TSB, shaking at 25°C and 150rpm for 1–7days, and melanin production in the broth was measured at OD_400_ using the standard synthetic melanin (Sigma-Aldrich, M8631) calibration curve method (Singh et al., 2018). Melanin was purified as previously described (El-Naggar and El-Ewasy, 2017). Briefly, the supernatant was centrifuged at 8,000rpm to remove the cells, followed by adjustment of the pH of the supernatant to 2.0 using 6M HCl; the samples were then allowed to stand for 4h and centrifuged at 8,000rpm to collect the precipitate. The melanin pellets were washed with distilled water three times and then centrifuged at 8,000rpm for 10min to obtain melanin. The purified melanin was freeze-dried for further use. *In vitro*-synthesized pyomelanin was produced by auto-oxidation of 10mM HGA (Sigma-Aldrich, H0751) solution at pH 10 with constant stirring for 3days as described by Schmaler-Ripcke et al. (2009).

### UV–Visible Spectroscopy and Fourier-Transform Infrared Spectroscopy

To analyze the pigment content of GR-TSA-9^T^, purified melanin was dissolved in 0.5M NaOH solution, and the absorption value was recorded in the wavelength range of 200–1000nm with a UV–visible spectrophotometer (Thermo Fisher Scientific, USA) by comparing it to a synthetic melanin standard. FT-IR spectroscopy was performed at the Center for Instrumental Analysis, Korea Basic Science Institute, Busan, Republic of Korea. The samples were pressed into disks under vacuum using a KBrpress. The FT-IR spectra of the KBr discs were recorded on an FT-IR Vertex 80v spectrophotometer (Bruker, USA). The spectra were read in the wavenumber region of 400–4000cm^−1^.

### Electrospray Ionization Mass Spectrometry

Mass spectrometry analysis was performed using an API 32000 mass spectrometer (AB SCIEX, USA). Samples were dissolved in 1M NaOH solution and then diluted with 50:50 methanol/water. The optimized mass spectrometry parameters were as follows: curtain gas, 10; spray voltage, 4200; ion source gas, 20psi; and flow rate, 10μl/ml. Full spectra were collected in the *m/z* range of 50–1800 in the positive ion mode.

### UVC Exposure and Sensitivity Tests

GR-TSA-9^T^ cells were cultured on TSA (non-melanized cells) and TSA with 10mg/ml l-tyrosine (melanized cells) at 25°C for 48h. The cells were washed twice with 1× phosphate-buffered saline. Next, 5×10^7^ cells were irradiated with UVC (254nm) at 450mJ/cm, and 10μl of aliquots from tenfold serial dilutions was spotted on TSA plates. All plates were incubated at 25°C for 3days. The experiments were repeated at least twice, with similar results.

## Results and Discussion

### Phenotypic and Biochemical Characterization

Strain GR-TSA-9^T^ grew well on LB, TSA, MA, NA, and R2A (optimum TSA and LB) but not on RC and PDA. Growth occurred at 10–37°C (optimal 25–30°C), in 0–1% (w/v) of NaCl (optimum 0%), and at pH 7.0–8.0. Cells were rod shaped (0.2–0.3μm in width and 0.9–2.6μm in length) with flagella (Supplementary Figure 1), gram negative, facultatively anaerobic, motile by swimming and twitching, catalase weakly positive, and oxidase positive. Other characteristics of strain GR-TSA-9^T^, which is different from closely related strains, are shown in Table 1.

**Table 1:** Differential physiological and biochemical comparison of strain GR-TSA-9^T^ and closely related type strains.

Characteristics	1	2	3	4	5	6^e^
Isolation source	Grape	Alkaline soil^a^	River^b^	Saline soil^c^	Soil	Fresh water
Colony color	Grayish white,	Grayish yellow	Ivory-colored^b^	White^c^	Grayish yellow^d^	White to creamy
Oxygen requirement	Facultatively aerobic	Aerobic^a^	Facultatively aerobic^b^	Aerobic^c^	Aerobic^d^	Aerobic
Colony shape	Circular, smooth, opaque, flat elevation	Circular, smooth, glistening, slightly convex^a^	Circular, convex, opaque^b^	Circular, smooth, slightly convex^c^	Circular, convex, glistening, sticky^d^	Smooth, raised, white to creamy
Catalase	w	+^a^	+^b^	+^c^	+^d^	+
Melanin production	+	+	−	+	+	−
Cell size (μm)	0.2–0.3×0.9–2.6	0.4–0. 6×1.0–3.0^a^	0.2–0. 5×1.0–3.0^b^	0.4–0.6×0.9–2.5^c^	0.3–0. 5×0.6–3.5^d^	0.5×1.0–4.0
Growth at:						
Temperature range (°C)	10–37 (25–30)	10–36 (30)	15–40	10–36 (28–30)	4–34 (25)	35
NaCl range (%, w/v)	0–1 (0)	0–2 (0)	0–2	0–3 (0)	0–1 (0)	7.0
pH range	7–8 (7)	6–9 (7–8)	6.0–8.0	5.5–11 (7–8)	6–9.5 (0.5–7.0)	−
API 20NE						
Nitrate reduction	−	−	−	+	−	−
β-galactosidase	+	−	−	−	−	ND
Glucose Assimilation	−	+	−	−	+	−
Maltose	−	+	−	−	+	−
Malate	−	+	+	−	−	−
Citrate	−	+	+	−	−	−
API ZYM						
Valine arylamidase	w	w	+	+	w	−
Cystine arylamidase	w	−	−	w	w	−
α-chymotrypsin	w	w	w	+	w	+
Acid phosphate	−	+	−	w	+	ND
Naphthol-AS-BI-phosphohydrolase	w	+	w	+	w	ND
α-glucosidase	+	+	+	+	w	−

Strains: 1, *Brevundimonas vitisensis* GR-TSA-9^T^; 2, *B. kwangchunensis* KCTC 12380^T^; 3, *B. fluminis* KCTC 72717^T^; 4, *B. viscosa* JCM 17426^T^; 5, *B. lenta* KCTC 12871^T^; and 6, *B. diminuta* AJ 2067^T^. All data are from the present study unless indicated otherwise. +, positive; w, weakly positive; −, negative; and ND, not detected.

a Yoon et al. (2006).

b Lee et al. (2020).

c Wang et al. (2012).

d Yoon et al. (2007).

e Segers et al. (1994).

### Phylogenetic Analyses

**Figure 1:**
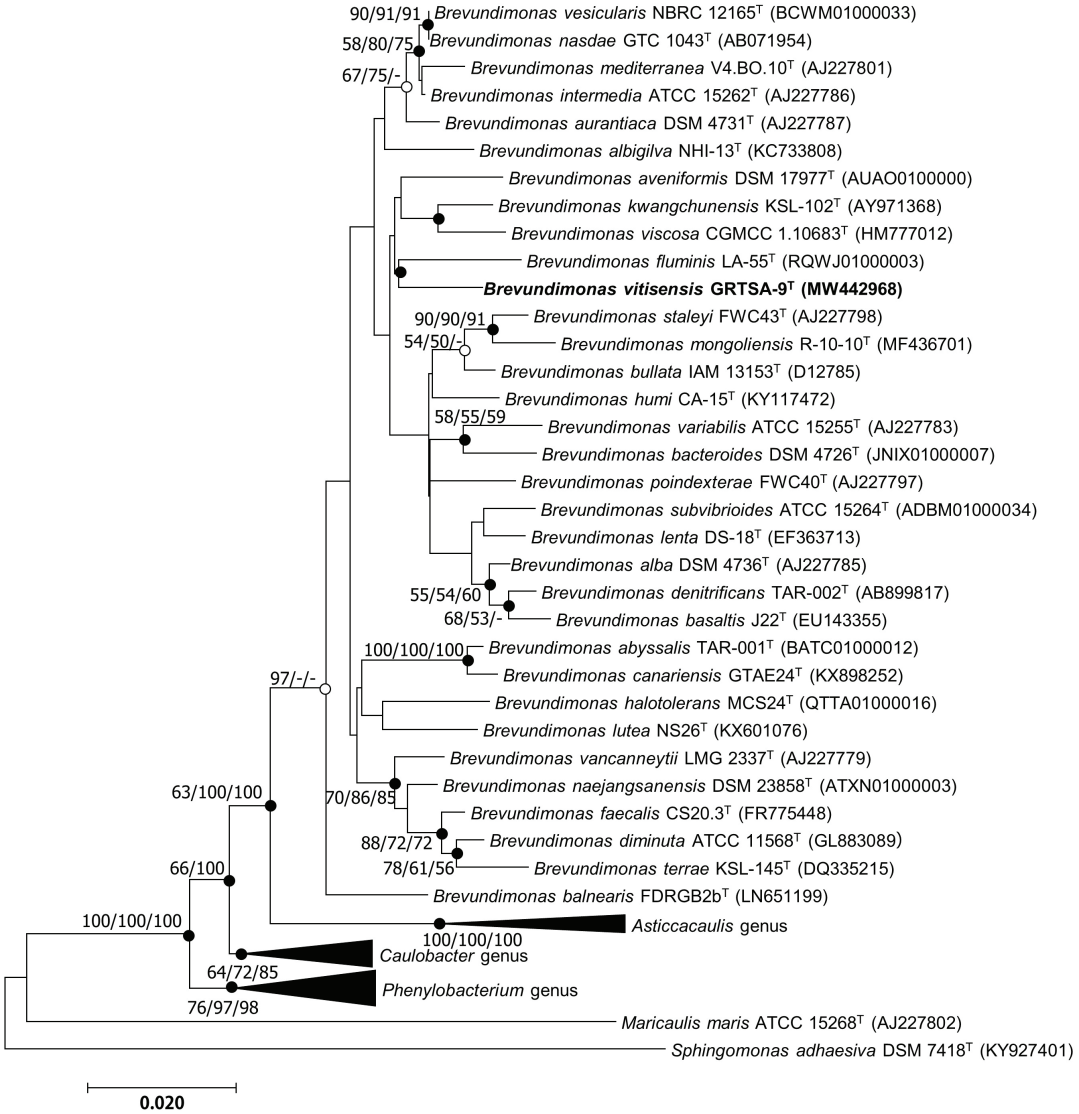
Neighbor-joining (NJ) algorithm phylogenetic tree based on 16S rRNA genes of strain GR-TSA-9^T^ and the members of the family *Caulobacteraceae*. Bootstrap values (>70%) calculated using the NJ, maximum likelihood (ML), and minimum evolution (ME) algorithms are shown. Filled circles on the nodes indicate that the relationships were also recovered by ML and ME algorithms, whereas open circles indicate nodes recovered by either ML or ME algorithms. Scale bar: 0.020 substitutions per nucleotide position.

The almost-complete 16S rRNA gene amplicon of strain GR-TSA-9^T^ contained 1394 nucleotides and has been submitted to GenBank (accession number: MW442968.1). Based on 16S rRNA gene sequence analysis, strain GR-TSA-9^T^ appeared to belong to the genus *Brevundimonas* and was closely related to *B. lenta* DS-18^T^ (97.9%), *B. kwangchunensis* KSL-102^T^ (97.8%), and *B. aurantiaca* DSM 4731^T^ (97.5%), whereas it shared less than 97.5% similarity with other related species. Based on the novel species recognition threshold of 98.6% (Kim et al., 2014), strain GR-TSA-9^T^ was regarded as a novel species in the genus *Brevundimonas*. A phylogenetic tree was reconstructed using the NJ, ME, and ML methods. The results of these analyses suggest that strain GR-TSA-9^T^ belongs to the genus *Brevundimonas*. The genera *Asticcacaulis*, *Caulobacter*, and *Phenylobacterium* formed independent and monophyletic branches (Figure 1). Moreover, *B. fluminis* LA-55^T^, *B. viscosa* CGMCC 1.10683^T^, *B. kwangchunensis* KSL-102^T^, *B. lenta* DS-18^T^, and *B. diminuta* AJ 2068^T^, the type species of genus *Brevundimonas*, were used to compare phenotypic properties and for chemotaxonomic analyses based on 16S rRNA similarity and phylogenetic tree analysis.

### Genome Properties and Genetic Relatedness

The genome of strain GR-TSA-9^T^ was assembled using the SMRT Portal (version 2.3) *de novo* assembler, which is a single circular chromosome of 2,976,716bp in size (the N50 value was 13,858bp), with a coverage of 389X. By comparing two copies of the 16S rRNA gene fragment from the whole-genome sequence, a contaminating DNA sequence was not present in the genome assembly. The genomic GC content of GR-TSA-9^T^ was 66.4%, which was similar to that of other type strains in the genus *Brevundimonas*. According to the PGAP annotation, there were 2940 protein-coding genes and 56 RNA genes, including two 5S rRNA genes, two 16S rRNA genes, two 23S rRNA genes, four ncRNAs, and 46 tRNA genes. Cluster orthologous group (COG) annotation results showed that the functional categories of most coding sequences were classified as unknown (28.6% of total assigned COGs), general function prediction only (9.1% of the total assigned COGs), and amino acid transport and metabolism (5.8% of the total assigned COGs; Supplementary Figure 2).

**Figure 2:**
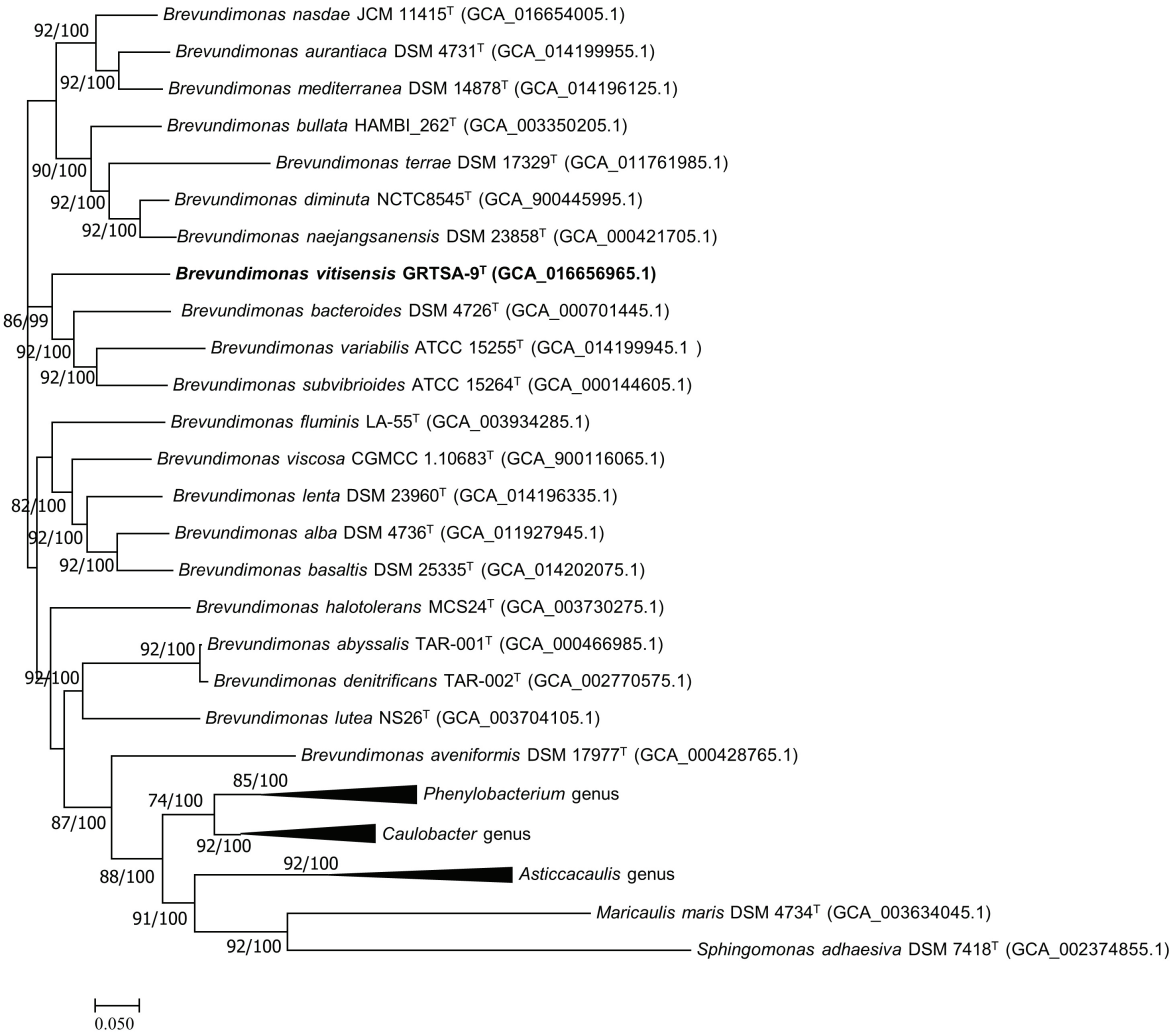
Maximum likelihood algorithm phylogenomic tree based on up-to-date bacterial core gene set (concatenated alignment of 92 core genes) showing the relationship between strain GR-TSA-9^T^ and the members of the family *Caulobacteraceae*. Gene support index (GSI, left) and bootstrap values (right) are indicated at the nodes. Scale bar: 0.050 substitutions per position.

The dDDH value between strain GR-TSA-9^T^ and the closest species *B. lenta* DS-18^T^ was 20.9%, while the ANI value was 76.7%. OrthoANI values based on the entire genome (Supplementary Figure 3) were 72.8–77.6% for the most closely related strains. Thus, strain GR-TSA-9^T^ was considered a distinct species of the genus *Brevundimonas*, considering the values obtained were significantly lower than the proposed dDDH (<70%) and ANI cutoff (95–96%) values for bacterial species delineation (Chun et al., 2018). The 92 core gene set-based phylogenetic tree also supported strain GR-TSA-9^T^ forming a phylogenetic lineage within the genus *Brevundimonas*, consistent with the 16S rRNA-based phylogenetic tree (Figure 2).

### Genome-Derived Features of Strain GR-TSA-9^T^

Genome annotation was performed using the RAST server and BlastKOALA to reconstruct the metabolic pathway, and the following sections were predicted from genome sequences.

#### Motility

Genes related to the motility subsystem were detected in the whole genome, including basal-body rod protein FlgB, FlgC, and FlgD; flagellar hook–basal body complex protein FliE; flagellar motor switch protein FliM and FliN; flagellar L-ring protein FlgH; flagellar biosynthesis protein FlhB; FlhR and FlhA; and flagellar motor rotation proteins MotA and MotB. These data are consistent with the motility test results.

#### Respiration

Ferredoxin reductase, one of the anaerobic respiratory reductases, was annotated in the whole genome, which explains the fact that the strain can grow under anaerobic conditions. Among all respiration-related genes, most are related to electron-donating and electron-accepting reactions, followed by ATP synthases, biotin synthesis, the quinone oxidoreductase family, biogenesis of cytochrome C oxidases, biogenesis of c-type cytochromes, soluble cytochromes, and functionally related electron carriers.

#### Stress Response

Osmotic stress-related genes encoding enzymes involved in the synthesis of osmoregulated periplasmic glucans and osmoregulation were found in the whole genome, and no gene related to heat shock, cold shock, or salt stress was observed in the whole genome. These results explain why the strain grows at a limited temperature and salinity range.

#### Glycerophospholipid and Glycerolipid Metabolism

The enzymes for glycerolipid and glycerophospholipid synthesis were found in the whole genome; genes encoding PG biosynthesis enzymes, such as phosphatidate cytidylyltransferase (EC 2.7.7.41) and CDP-diacylglycerol-glycerol-3-phosphate 3-phosphatidyltransferase (EC 2.7.8.5), and phosphatidylcholine biosynthesis enzymes, such as glycerol-3-phosphate dehydrogenase (EC 1.1.5.3) and glycerol-3-phosphate dehydrogenase [NAD(P)+] (EC 1.1.1.94), were also annotated in the whole genome.

### Chemotaxonomic Characterization

The cellular fatty acid (>1%) composition of strain GR-TSA-9^T^ and five closely related strains is listed in Table 2; the most abundant fatty acids of strain GR-TSA-9^T^ were C_16:0_ (14.2%) and summed featured 8 (65.6%). These results are consistent with those of closely related species within the genus *Brevundimonas*. However, the amount of some components, such as summed featured 3, C_17:1_ w8c, and C_18:1_ w7c 11-methyl, differs from the reference strains. The respiratory quinone detected in strain GR-TSA-9^T^ was Q-10, which was consistent with the genus *Brevundimonas*. The polar lipid profile of strain GR-TSA-9^T^ consisted of phosphoglycolipids, phosphatidylglycerol, 1,2-di-*O-*acyl-3-O-[d-glucopyranosyl-(1→4)-α-d-glucopyranuronosyl]glycerol, and unidentified lipids (L1, L2, and L4; Supplementary Figure 4), and lipid spots different from other closely related strains suggested that strain GR-TSA-9^T^ represents a novel species in the genus *Brevundimonas*.

**Table 2:** Cellular fatty acid profiles (>1%) of strain GR-TSA-9^T^ and its closely related species.

Fatty acid	1	2	3	4	5	6^a^
**Saturated**						
C_14:0_	0.3	1.9	1.8	0.4	1.6	1.5±0.2
C_15:0_	ND	ND	ND	ND	ND	1.3±0.5
C_16:0_	**14.2**	**19.8**	**15.6**	**11.5**	**18.5**	**30.4±2.2**
C_17:0_	1.0	0.2	5.7	7.7	1.0	1.2±0.5
C_18:0_	0.6	0.3	0.2	0.6	1.0	ND
**Hydroxy**						
3OH-C_12:1_	0.3	0.5	2.3	1.2	0.1	ND
3OH-C_12:0_	3.2	2.5	0.9	0.8	3.1	2.6±0.4
**Unsaturated**						
C_15:1_ w8c	ND	ND	2.0	0.4	ND	ND
C_16:1_ w7c	ND	ND	ND	ND	ND	2.2±0.7
C_17:1_ w6c	1.9	0.3	5.1	6.0	0.8	ND
C_17:1_ w8c	2.4	0.7	**11.9**	**15.1**	2.2	ND
C_19:0_ cyclo w8c	ND	ND	ND	ND	ND	**10.1±3.7**
C_18:1_ w7c 11-methyl	4.5	7.1	**11.3**	0.6	4.5	ND
**Summed feature** ^*^						
2	1.7	ND	ND	ND	ND	ND
3	3.0	**12.1**	6.0	1.3	8.1	ND
7	ND	ND	ND	ND	ND	**45.3±6.5**
8	**65.6**	**53.5**	**36.1**	**51.3**	**58.3**	ND

Values are percentages of total fatty acids. ND, not detected. The major components (>10%) are shown in bold. Strains: 1, *Brevundimonas vitisensis* GR-TSA-9^T^; 2, *B. kwangchunensis* KCTC 12380^T^; 3, *B. fluminis* KCTC 72717^T^; 4, *B. viscosa* JCM 17426^T^; 5, *B. lenta* KCTC 12871^T^; and 6, *B. diminuta* AJ 2067^T^. All the data were obtained from the present study unless indicated otherwise.

* Summed features are groups of two or three fatty acids that cannot be separated by GLC with the MIDI System. Summed feature 2 contains C_14:0_-3OH or/and C_16:1_ iso. Summed feature 3 contains C_16:1_ w6c or/and C_16:1_ w7c. Summed feature 7 contains C_18:1_ w7c, C_18:1_ w9t, C_18:1_ w12t. Summed feature 8 was listed as C_18:1_ w7c or/and C_18:1_ w6c.

a Segers et al. (1994).

### Pigment Production

**Figure 3:**
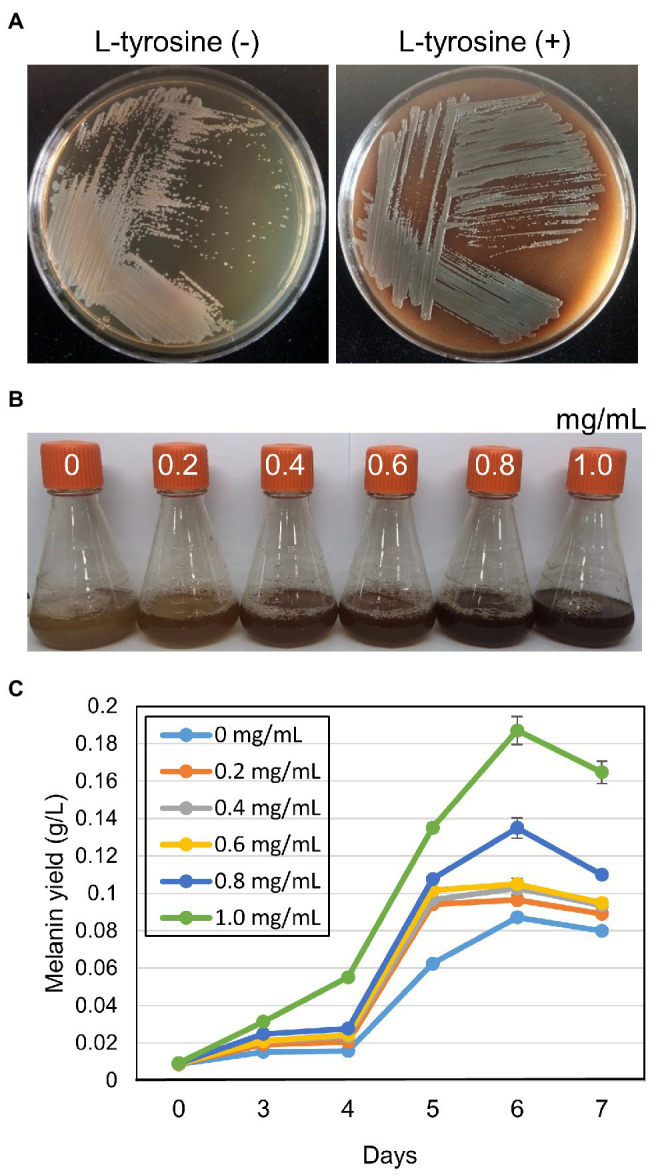
Melanin-like pigment production of strain GR-TSA-9^T^ on tryptic soy agar (TSA) medium. **(A)** Melanin-like pigment production of strain GR-TSA-9^T^ on TSA without and with L-tyrosine participation; dark brown pigment was produced on the L-tyrosine medium for 5days. **(B,C)** Effect of L-tyrosine concentration on melanin production in TSB medium. The graph represents the average of the results from three replicate measurements.

Strain GR-TSA-9^T^ was considered a potential melanin producer on TSA medium and can produce a higher amount of melanin in l-tyrosine-containing medium than without l-tyrosine (Figures 3A,B). On TSA with l-tyrosine, the synthesis of brown pigment was initiated at 3 d and the pigment increased to brown, dark brown, and black-brown at 4, 5, and 6days, respectively. Melanin production in TSB was observed after 3days of shaking, and at 6days, the highest yield of melanin (0.19g/L) was obtained (Figure 3C).

### Melanin Biosynthetic Pathway *via* Genomic Annotation

**Figure 4:**
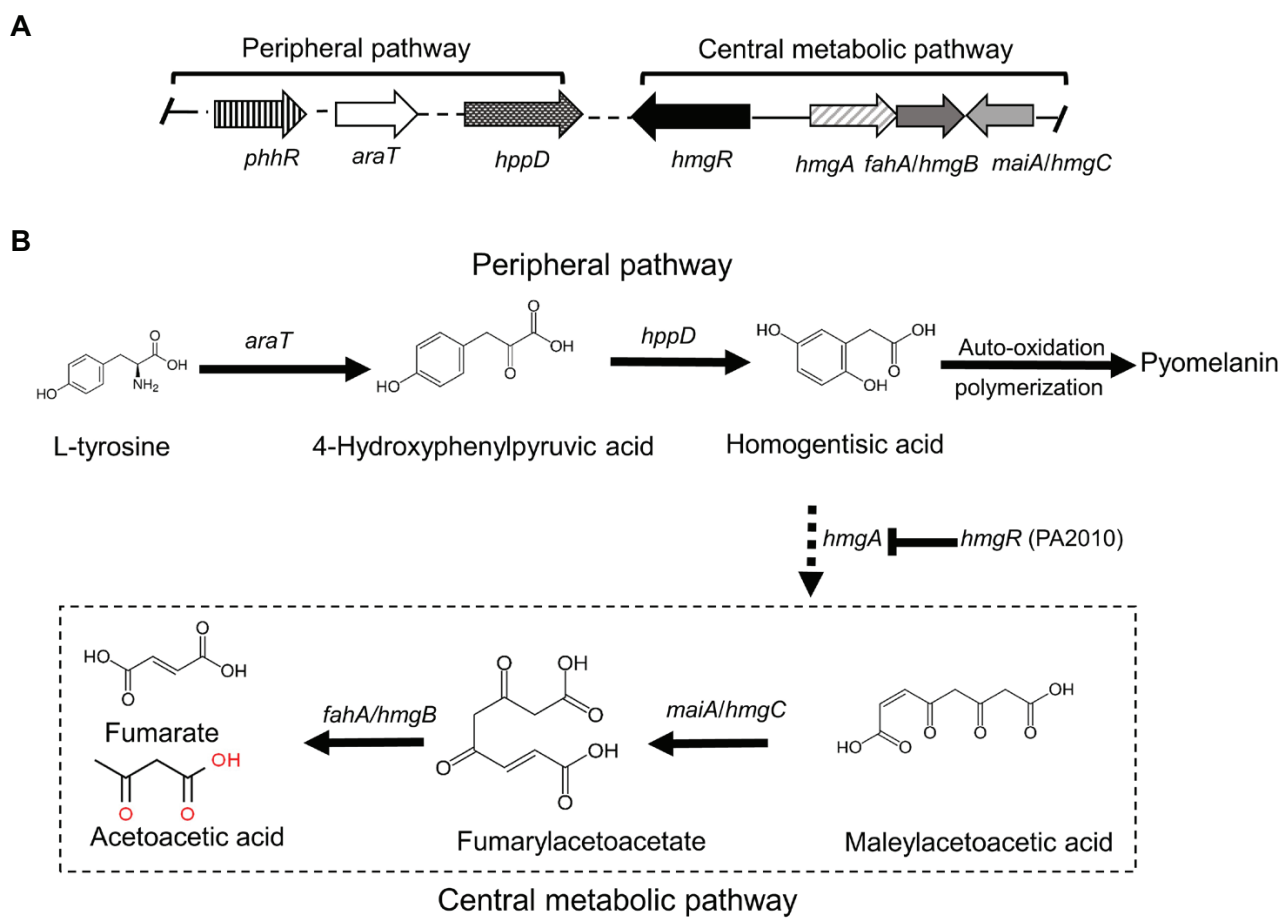
Proposed pathway and gene clusters of pyomelanin biosynthesis in strain GR-TSA-9^T^ (modified from Schmaler-Ripcke et al., 2009). **(A)** Gene cluster organization and expression of pyomelanin biosynthesis, homogentisate, and central pathways. **(B)** Pyomelanin biosynthesis and regulation pathway. The enzymes involved are as follows: *phhR* (JIP62_13420; JIP62_13620; JIP62_02890; and JIP62_0119), sigma-54-dependent Fis family transcriptional regulator; *araT* (JIP62_09170), aromatic amino acid aminotransferase (EC 2.6.1.57); *hppD* (JIP62_07560), hydroxyphenylpyruvate dioxygenase (EC 1.13.11.27); *hmgR* (JIP62_11565; JIP62_04630; and JIP62_01885), TetR family member transcriptional regulator; *hmgA* (JIP62_08245), homogentisate dioxygenase; *hmgC*/*maiA* (JIP62_08415), maleylacetoacetate isomerase; and *fahA*/*hmgB* (JIP62_RS07590; JIP62_RS08410; and JIP62_RS01250), fumarylacetoacetate hydrolase.

Strain GR-TSA-9^T^ was found to produce dark brown melanin-like pigment in TSA medium containing l-tyrosine (Figure 3A), suggesting that l-tyrosine is a precursor for melanin biosynthesis in strain GR-TSA-9^T^. In addition, it has been reported that *Brevundimonas* sp. SGJ produces L-DOPA melanin (Surwase et al., 2012a,b, 2013). However, we failed to identify annotated tyrosinases (EC 1.14.18) and laccases (EC 1.10.3.2) with the PGAP and RAST annotation pipelines. These data indicate that melanin synthesis in strain GR-TSA-9^T^ may not occur *via* the L-DOPA melanin biosynthetic pathway. Some interesting features of strain GR-TSA-9^T^ were observed HGA-melanin biosynthesis pathway in the whole genome (Figure 4). According to the HGA-melanin synthesis pathway described previously (Hunter and Newman, 2010; Drewnowska et al., 2015; Wang et al., 2015), all genes responsible for the HGA melanin synthesis pathway were predicted from the whole genome as follows: *araT* encoding amino acid aminotransferase (E.E.2.6.1.57) converts tyrosine to 4-hydroxyphenylpyruvic acid (HPP), and *hppD* encoding 4-hydroxyphenylpyuvate dioxygenase (EC 1.13.11.27) catalyzes conversion of 4-HPP to HGA. Then, the produced HGA is converted to pyomelanin through auto-oxidation and spontaneous polymerization. Homogentisic acid is catabolized by a central metabolic pathway that involves three enzymes, homogentisate dioxygenase (*hmgA*; EC 1.13.11.5), fumarylacetoacetate hydrolase (*hmgB*/*fahA*), and maleylacetoacetate isomerase (*maiA*/*hmgC*), and then, final products are fumarate (EC 4.2.1.2) and acetoacetate (Arias-Barrau et al., 2004). The pyomelanin production results from a defect in the catabolism pathway (Fonseca et al., 2020). When *hmgA* levels are low (e.g., gene mutation, deletions, or overexpression of *hmgR*, HGA accumulates and secretes out of cells, and it lead to pyomelanin though HGA auto-oxidation and self-polymerization. (Arias-Barrau et al., 2004; Rodríguez-Rojas et al., 2009; Wang et al., 2011; Fonseca et al., 2020). In the whole genome of strain GR-TSA-9^T^, TetR family member *hmgR* (PA2010), which was previously shown to bind directly to the *hmgA* promoter and repress *hmgA* expression, blocks the central pathway and finally accumulates homogentisic acid and produces pyomelanin (Figure 4A). In strain GR-TSA-9^T^, the gene *hppD* (EC 1.13.11.27) encodes the 4-hydroxyphenylpyruvate dioxygenase, *araT* (EC 2.6.1.57), and fumarate hydratase (EC 4.2.1.2), which share 62.5, 48.6, and 64% identity with the corresponding protein of *Pseudomonas aeruginosa* PAO1 (AE004091.2), which was found to produce pyomelanin through the HGA pathway (Bolognese et al., 2019). It is possible that strain GR-TSA-9^T^ produces pyomelanin through HGA melanin, which encodes *araT*, *phhR* encoding σ^54^-dependent transcriptional activator, and *hppD*, constituting a linear pathway for converting phenylalanine to HGA, which is widely conserved in the genus *Pseudomonas* (Arai et al., 1980; Arias-Barrau et al., 2004; Rodríguez-Rojas et al., 2009; Orlandi et al., 2015; Hocquet et al., 2016; Zeng et al., 2017; Kurian and Bhat, 2018; Bolognese et al., 2019). This suggests that encoding the most critical enzymes 4-hydroxyphenylpyruvate dioxygenase (EC 1.13.11.27), *araT* (EC 2.6.1.57), and other enzymes such as *hmgR* annotated in the whole genome demonstrated that the HGA pathway is the melanogenic pathway in strain GR-TSA-9^T^ (Figure 4B). In addition, we found brown or black pigments in some of the most closely related strains such as *B. Kwangchunensis* KCTC 12380^T^, *B. viscosa* JCM 17426^T^, and *B. lenta* KCTC 12871^T^ strains, but not in *B. fluminis* KCTC 72717^T^ and *B. diminuta* AJ2067^T^ (Table 1). To determine whether the HGA pathway is specific to strain GR-TSA-9^T^ or common to the genus *Brevundimonas*, we investigated the important enzyme-coding genes, such as *hppD*, *araT*, and *hmgA*, involved in the HGA melanin synthesis pathway. Our bioinformatic analysis results showed that the key genes related to the HGA-pathway are widely distributed in the genus *Brevundimonas*, including *Brevundimonas naejangsanensis* BRV3, *Brevundimonas subvibrioides* ATCC 15264^T^, *Brevundimonas abyssalis* TAR-001^T^, and *Brevundimonas viscosa* CGMCC 1.10683^T^. However, the genes *phhR* and *araT* could not identify from whole genome of *B. fluminis* KCTC 72717^T^. Although the presence of HGA-melanin-related genes is necessary but insufficient for pyomelanin production (if it cannot block and convert into a central pathway), some *Brevundimonas* species cannot produce pyomelanin because of the incomplete pathway. Based on these analyses, we suggest that the brown-to-black pigment production in many species in the genus *Brevundimonas* is because of the production of pyomelanin *via* the HGA pathway rather than the L-DOPA pathway.

### Pigment Characterization

**Figure 5:**
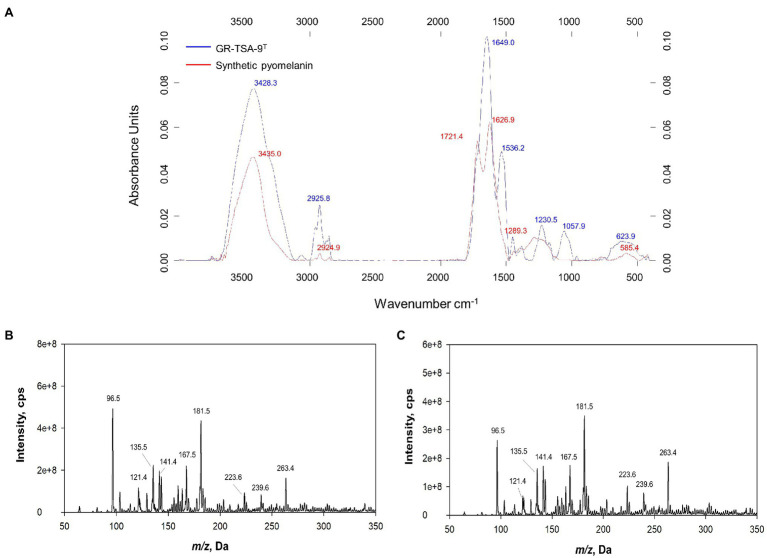
Analysis of pyomelanin from strain GR-TSA-9^T^. **(A)** FT-IR spectrum overlay from synthetic pyomelanin (red line) and purified melanin from strain GR-TSA-9^T^ (blue line). Electrospray ionization mass spectrometry (ESI-MS) spectra of synthetic pyomelanin **(B)** and purified bacterial melanin **(C)**. Nine main peaks (*m/z* ration: 96.5, 121.4, 135.5, 141.4, 167.5, 181.5, 223.6, 239.6, and 263.4) are evident in the pigment of synthetic and bacterial melanin.

The wavelength of maximum absorbance was scanned in the range of 200–1000nm. The wavelength of maximum absorbance of the extracted pigment from the culture supernatant and synthetic melanin standard was observed at 210nm (Supplementary Figure 5), which is a typical feature of melanin (ranges between 196 and 300nm; Surwase et al., 2013; Zeng et al., 2017). The FT-IR spectra were analyzed to confirm that the extracted bacterial pigment was pyomelanin type of melanin. The FT-IR spectra of the extracted bacterial pigment and synthetic pyomelanin standard are shown in Figure 5A. The spectra were recorded at 4000–400cm^−1^ using an FT-IR spectrophotometer. Both spectra revealed a broad absorption peak at 3650–3100cm^−1^, corresponding to the ▬OH group and ▬NH group. The peaks at 3000–2900 and 1250–1200cm^−1^ were attributed to C▬H groups. The strong absorption peak observed at 1450–1770cm^−1^ was ascribed to the C〓C and C〓O groups. The peak between 1250 and 1200cm^−1^ corresponds to the N▬H group and C▬N (secondary amine). The high degree of resemblance in the main absorption peaks indicated that the pigment isolated from strain GR-TSA-9^T^ was pyomelanin. Synthetic pyomelanin and purified bacterial melanin were analyzed by ESI-MS. In addition, nine prominent peaks were observed in the mass spectrum of synthetic pyomelanin with *m/z* values of 96.5, 121.4, 135.5, 141.4, 167.5, 181.5, 223.6, 239.6, and 263.4 (Figure 5B). These nine peaks were also detected in the mass spectrum of purified bacterial melanin (Figure 5C). In particular, *m/z* 141.4 and 263.4 peaks were also detected in a previous report (Singh et al., 2018). These results suggest that the purified bacterial melanin is similar to synthetic pyomelanin.

### UVC Tolerance

**Figure 6:**
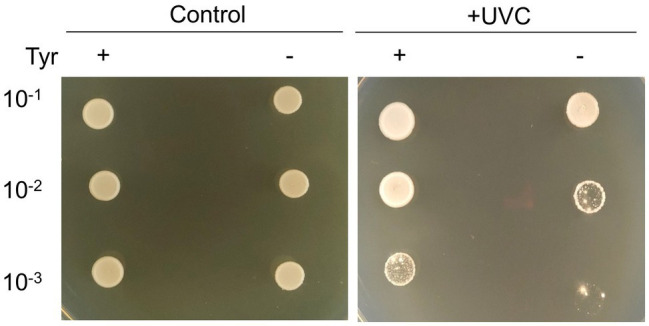
Photoprotective properties of strain GR-TSA-9^T^. Non-melanized and melanized GR-TSA-9^T^ cells were irradiated with 450mJ/cm^2^ UVC and then spotted in serial dilutions on TSA media for 3days.

To test the photoprotective property of bacterial melanin, cells were irradiated with short-wavelength UVC, which is a damaging type of UV radiation. UVC is absorbed by DNA, resulting in the formation of pyrimidine adducts and strand breaks (Rastogi et al., 2010). The cultured cells on TSA (non-melanized cells) and TSA with 10mg/ml l-tyrosine (melanized cells) were irradiated with UVC. The melanized cells grew better than the non-melanized cells after UVC irradiation (Figure 6). This finding suggests that cells grown in melanin-containing media showed significantly higher resistance to UV radiation than those grown on TSA medium alone.

## Conclusion

Together, the results, including phenotypic, physiological, phylogenetic, and biochemical analyses, indicate that strain GR-TSA-9^T^ represents a novel member of the genus *Brevundimonas*. The phylogenetic relationships of strain GR-TSA-9^T^ and other type strains of the genus *Brevundimonas* were revealed by phylogenetic trees based on 16S rRNA and core gene sets from the whole-genome sequence. The similarities in physiological characteristics and differences in biochemical characteristics with the closely related strains suggest that GR-TSA-9^T^ is a new member of the genus *Brevundimonas*. The present study is the first to report a type strain in the genus *Brevundimonas*, which produces pyomelanin. This study provides new insights into the formation and regulation mechanism of melanin, provides the exploration potential of strain GR-TSA-9^T^, and paves the way for industrial production in the future. The isolated melanin may have potential applications in the cosmetic and pharmaceutical industries.

### Description of *Brevundimonas vitisensis* sp. nov.

*Brevundimonas vitisensis* (vi’ tis.ne.sis. N.L. fem. *Vitis*, the generic name of grapevine, refers to the source from which the bacteria were isolated).

Colonies are grayish white, circular, smooth, and opaque (2–4mm in diameter) after growth on TSA medium at 25°C for 3days. Brown pigment is found to be a common attribute of melanin production, with optimal growth occurring at 25–30°C and pH 7.0 and in 0% (w/v) NaCl. Additionally, strain growth occurs on NA, MA, LB, TSA, and R2A (optimal TSA and LB) but not on PD and RC media. Cells are facultatively anaerobic, gram negative, rod shaped, (0.2–0.3μm×0.9–2.6μm), motile, catalase weak positive, and oxidase positive. In the assay with the API 20NE strips, reactions for esculin hydrolysis and β-galactosidase are positive, whereas for other substrates are negative. The production of acid is only from salicin. In the API ZYM strips, alkaline phosphatase, esterase (C4), esterase lipase (C8), leucine arylamidase, trypsin, β-glucosidase, and α-glucosidase are positive and valine arylamidase, cystine arylamidase, α-chymotrypsin, and naphthol-AS-BI-phosphohydrolase are weak positive. The major fatty acids are C_16:0_ (14.2%) and summed feature 8 (65.6%). The respiratory quinone detected in strain GR-TSA-9^T^ is Q-10, while the polar lipid profile of strain GR-TSA-9^T^ consisted of phosphoglycolipids, phosphatidylglycerol, 1,2-di-*O-*acyl-3-O-[d-glucopyranosyl-(1→4)-α-d-glucopyranuronosyl]glycerol, and unidentified lipids (L1, L2, and L4).

The type strain is *B. vitisensis* GR-TSA-9^T^ (=KCTC 82386^T^=CGMCC 1.18820^T^), isolated from surface-sterilized grapes from Jeongeup, South Korea.

The accession numbers for 16S rRNA and the whole genome of strain GR-TSA-9^T^ are MW442968.1 and CP067977.

## Data Availability Statement

The datasets generated for this study are available in the NCBI database. The accession numbers for 16S rRNA and the whole genome of strain GR-TSA-9T are as follows: GenBank MW442968.1 and CP067977.

## Author Contributions

JL and CK contributed to the conception, supervised the project, and edited the manuscript. LJ, DJ, and JK collected the data and carried out the experiment. LJ and JL wrote the original manuscript and revised the manuscript. YP, JS, JHL, CL, and JP helped with data curation. LJ, DJ, JK, CL, YP, JS, JHL, JP, CK, and JL provided critical feedback of the manuscript. All authors contributed to the article and approved the submitted version.

## Funding

This work was supported by the Korea Institute of Planning and Evaluation for Technology in Food, Agriculture and Forestry (IPET) through Agricultural Machinery/Equipment Localization Technology Development Program, the Ministry of Agriculture, Food and Rural Affairs (MAFRA; 321057051HD020), and the KRIBB Research Initiative Program (KGM5282122).

## Conflict of Interest

The authors declare that the research was conducted in the absence of any commercial or financial relationships that could be construed as a potential conflict of interest.

## Publisher’s Note

All claims expressed in this article are solely those of the authors and do not necessarily represent those of their affiliated organizations, or those of the publisher, the editors and the reviewers. Any product that may be evaluated in this article, or claim that may be made by its manufacturer, is not guaranteed or endorsed by the publisher.

## Footnotes

^1^ https://lpsn.dsmz.de/genus/brevundimonas

^2^ http://www.ezbiocloud.net

^3^ https://www.ezbiocloud.net/tools/contest16s

^4^ http://ggdc.dsmz.de/distcalc2.php

^5^ https://www.ezbiocloud.net/tools/ani
