# Supplementary material for: Pyomelanin-Producing Brevundimonas vitisensis sp. nov., Isolated From Grape (Vitis vinifera L.)
Source: Front Microbiol. 2021 Oct 14;12:733612. doi: 10.3389/fmicb.2021.733612 (PMC8551962; doi:10.3389/fmicb.2021.733612)
Supplement: Supplementary file 1 [file Data_Sheet_1.pdf]

Supplementary Material

Supplementary Figures

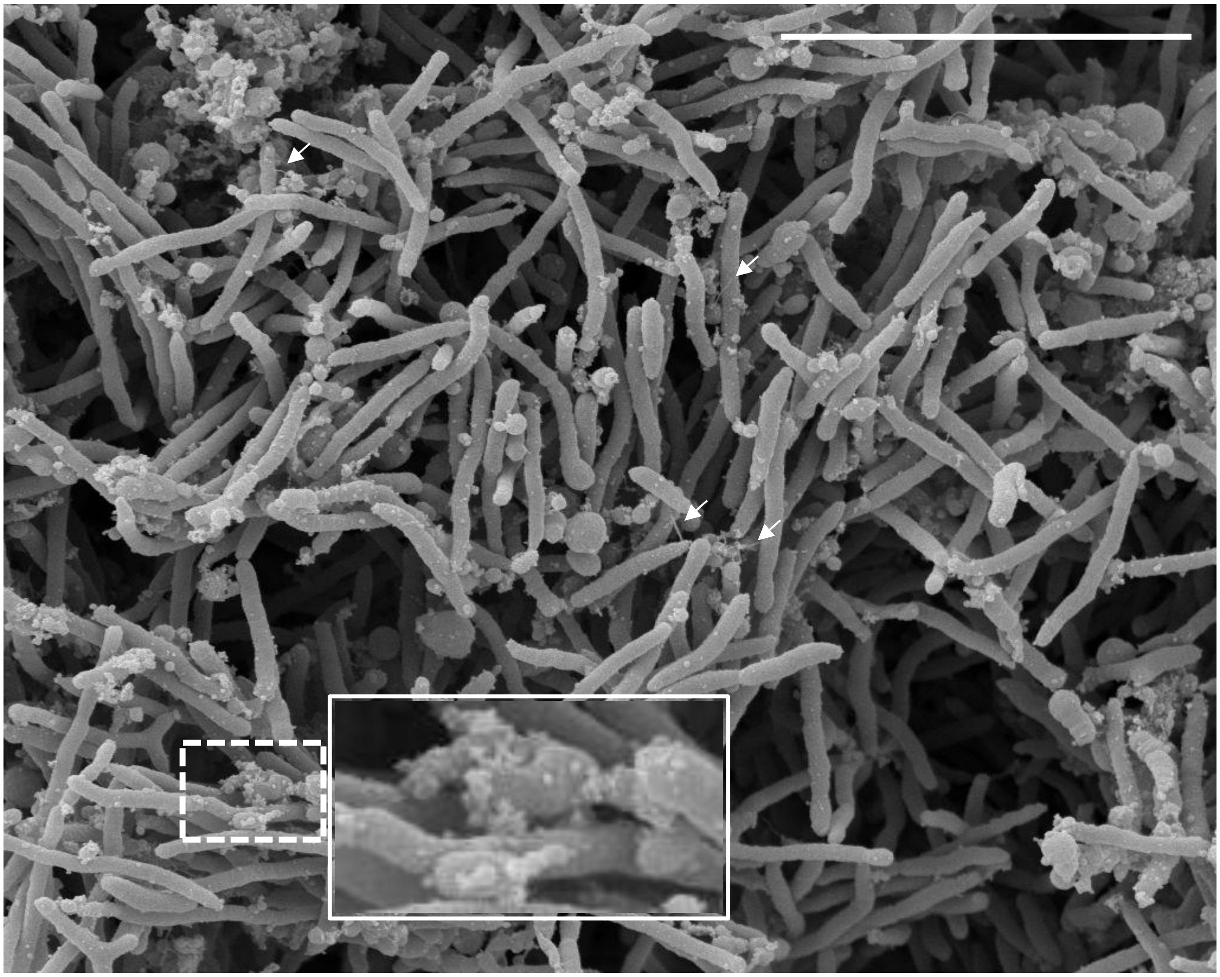

Supplementary Figure 1. Scanning electronic micrograph of strain GR-TSA-9^T. Inset showing the zoomed in image of melanin. Arrows show the flagella. Scale bar = 5 μm.

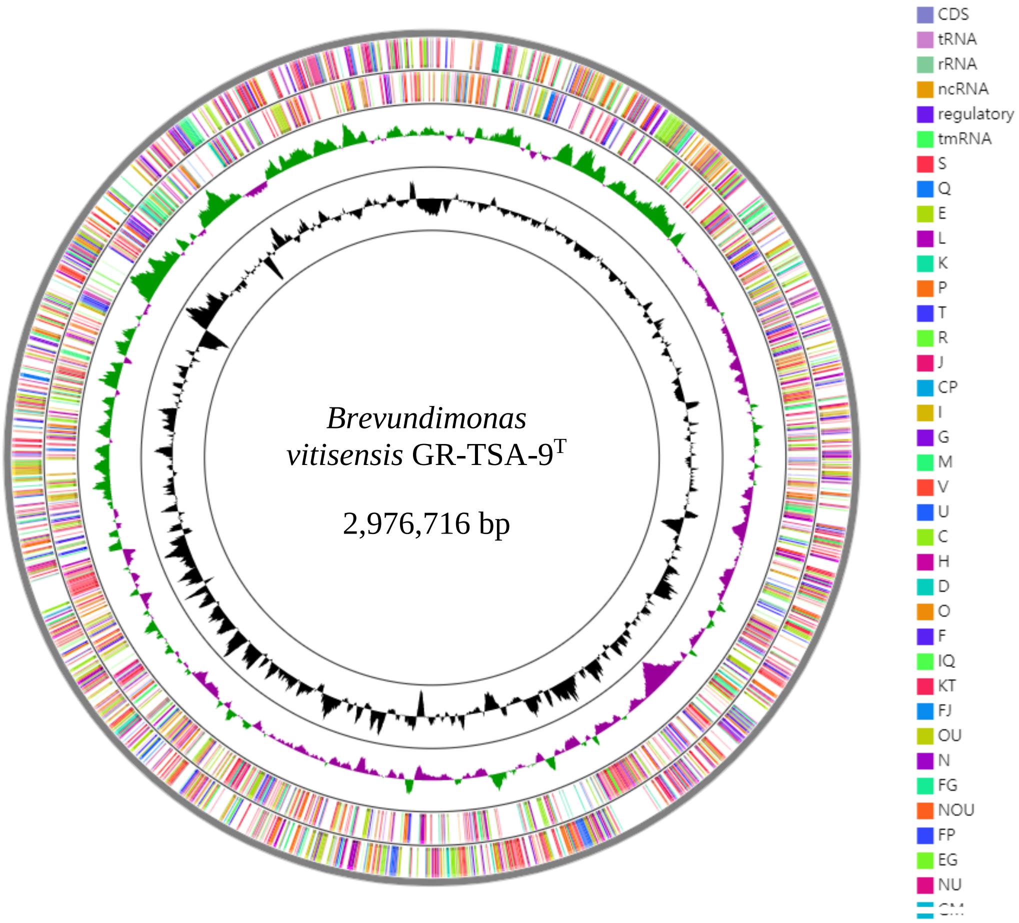

Supplementary Figure 2. Map of the GR-TSA-9^{T} genome. Marked characteristics are shown from the outside to the center. Rings 1 and 2 show cluster orthologous group (COG) annotation in the forward and reverse directions, respectively. Ring 3 showed the GC skew while ring 4 shows the G+C % content plot. The COG categories are A, RNA processing and modification; B, chromatin structure and dynamics; C, energy production and conversion; D, cell cycle control, cell division, and chromosome partitioning; E, amino acid transport and metabolism; F, nucleotide transport and metabolism; G, carbohydrate transport and metabolism; H, coenzyme transport and metabolism; I, lipid transport and metabolism; J, translation, ribosomal structure, and biogenesis; K, transcription; L, replication, recombination, and repair; M, cell wall/membrane/envelope biogenesis; N, cell motility; O, post-translational modification, protein turnover, chaperones; P, inorganic ion transport and metabolism; Q, secondary metabolite biosynthesis, transport, and catabolism; R, general function prediction only; S, function unknown; T, signal transduction mechanisms; U, intracellular trafficking, secretion, and vesicular transport; V, defense mechanisms; W, extracellular structures; X, mobilome: prophages, transposons; and Z, cytoskeleton.

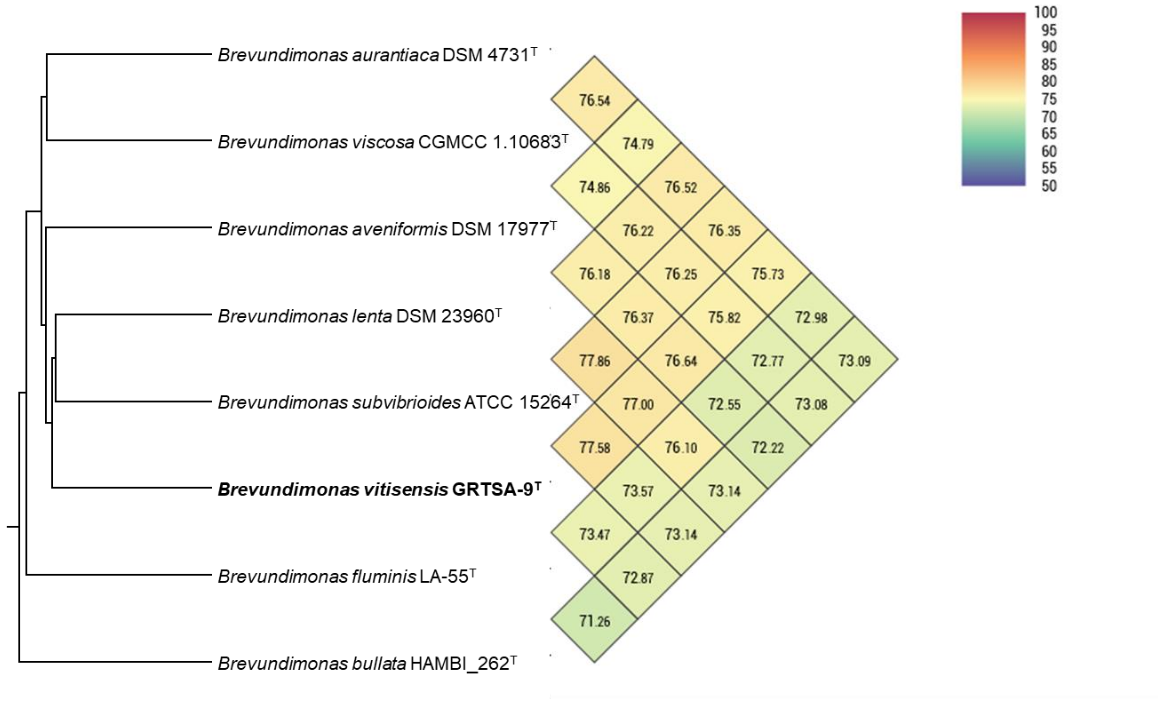

Supplementary Figure 3. Heatmap generated with OrthoANI value calculated using OAT software, comparing GR-TSA-9^{T} and closely related strains in *Brevundimonas*. The color code indicates the closest species with red and the farthest with green. Unweighted pair group method with arithmetic mean (UPGMA) dendrogram based on the OrthoANI values of eight species was listed on the left.

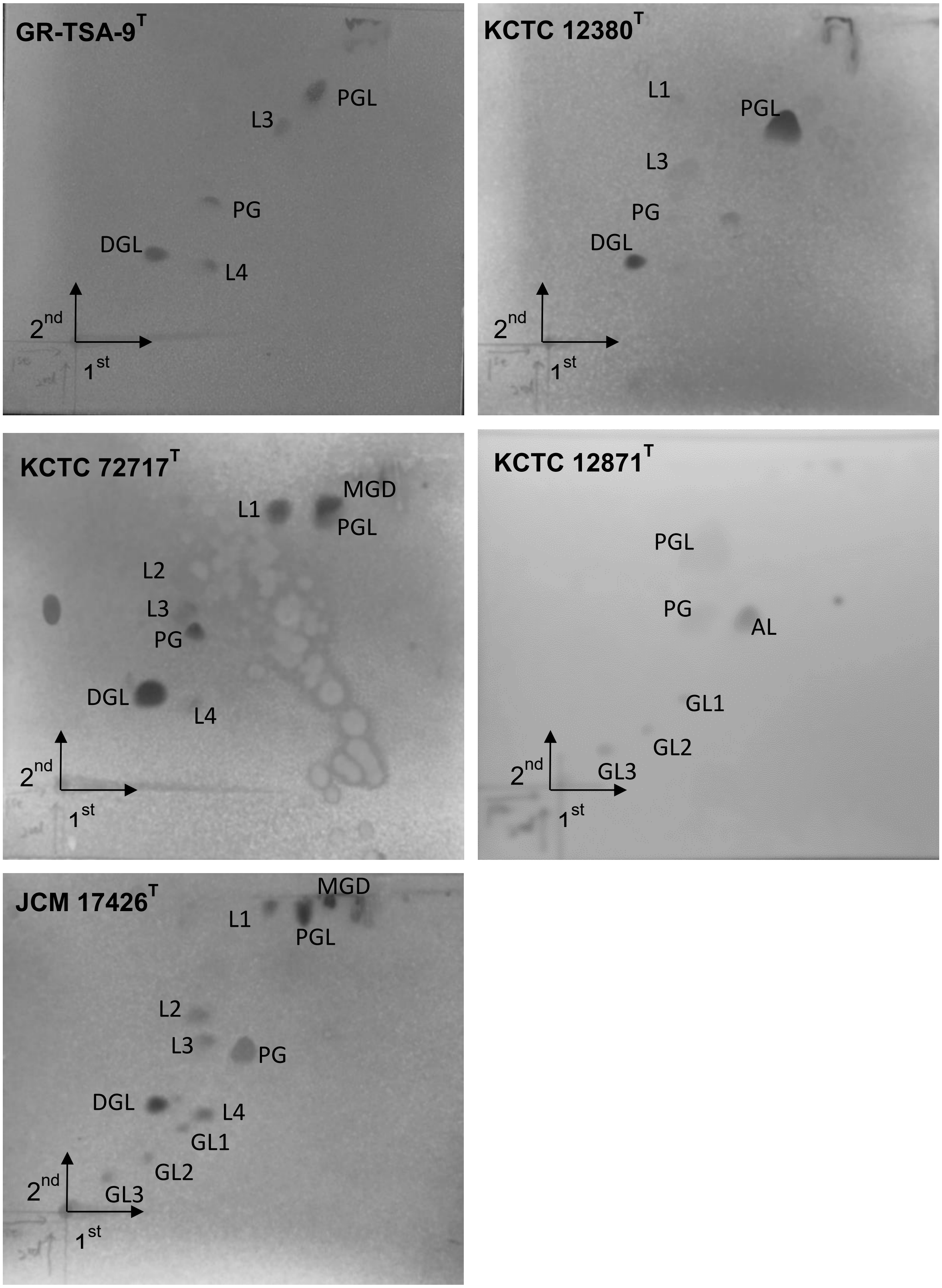

Supplementary Figure 4. Polar lipid profile of strain GR-TSA-9^{T} and related type strains. *Brevundimonas vitisensis* GR-TSA-9^{T}; *B. kwangchunensis* KCTC 12380^{T}; *B. fluminis* KCTC 72717^{T}; *B. lenta* KCTC 12871^{T}; *B. viscosa* JCM 17426^{T}.

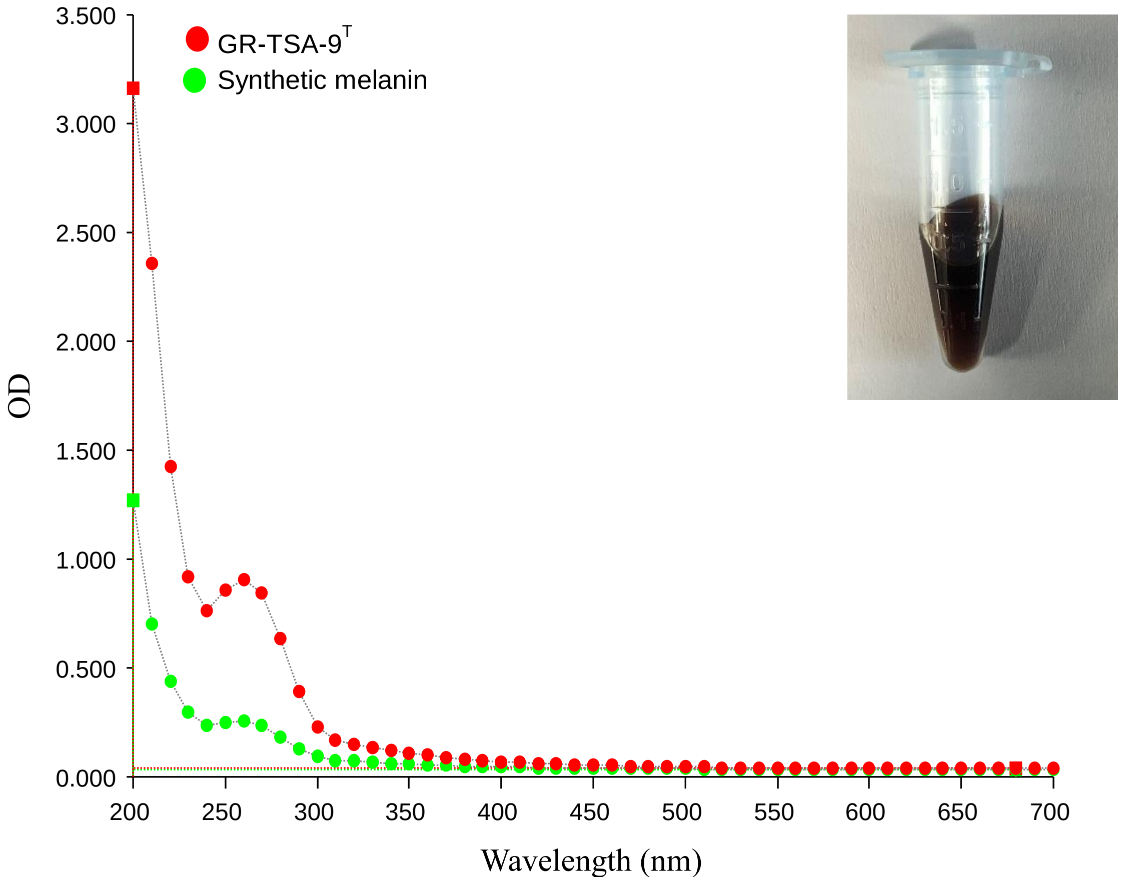

Supplementary Figure 5. UV-visible absorbance spectrum (200–700 nm) of purified melanin and synthetic melanin.
